# A spatiotemporal dataset of invasive *Anadara kagoshimensis* and *Anadara transversa* in the Adriatic Sea

**DOI:** 10.1038/s41597-025-05860-6

**Published:** 2025-09-29

**Authors:** Marina Chiappi, Cristina Di Muri, Ernesto Azzurro, Francesca Luzi, Ilaria Rosati, Marija Despalatović, Ivan Cvitković, Giuseppe Scarcella

**Affiliations:** 1 Department of Biological, Geological and Environmental Sciences (BIGEA), University of Bologna (UNIBO), Bologna, Italy; 2 National Research Council (CNR), Institute for Biological Resources and Marine Biotechnologies (IRBIM), Ancona, Italy; 3 NBFC, National Biodiversity Future Center, Palermo, Italy; 4 National Research Council (CNR), Research Institute on Terrestrial Ecosystems (IRET), Lecce, Italy; 5 LifeWatch Italy, Lecce, Italy; 6 Institute of Oceanography and Fisheries (IOF), Split, Croatia

**Keywords:** Invasive species, Biodiversity

## Abstract

In response to the growing demand for accurate spatial and temporal information on the abundance and distribution of invasive species as required by EU regulations, data on two invasive bivalves have been collected annually through beam trawl surveys in the Adriatic Sea, as part of an international, fishery-independent monitoring programme. The compiled dataset comprises 1,998 records of *Anadara kagoshimensis* and *Anadara transversa* located in the Italian and international waters of the Northern and Central Adriatic Sea, and collected from 2008 to 2023. The records from this dataset represent georeferenced information on detections/non-detections including abundance and biomass information per sampled sites. This initiative highlights the potential of leveraging existing spatiotemporal data on invasive species to support their commercial harvesting and inform sustainable management practices, ultimately helping to mitigate their impact on native ecosystems.

## Background & Summary

Invasive alien species are one of the key targets of the EU Biodiversity Strategy for 2030^1^ as they are considered drivers of biodiversity loss alongside other stressors (i.e., land and sea-use change, overexploitation, climate change, and pollution). The strategy promotes monitoring of invasive alien species through enhanced implementation of the Regulation (EU) 1143/2014^2^ which aims at developing an adequate knowledge base to address the issue by adopting surveillance systems based on both target and general biodiversity surveys. The bivalves *Anadara kagoshimensis* (Tokunaga, 1906) and *Anadara transversa* (Say, 1822) have been identified as two of the 100 most detrimental invasive species in the Mediterranean Sea^3^. In its native range, *Anadara kagoshimensis* has a wide geographical distribution, extending from the Central Indian Ocean to the Western Pacific, encompassing regions such as India, Sri Lanka, Indonesia, Korea, China, Japan, and Northern Australia^4^. The species was first documented in the Mediterranean Sea within the Italian Adriatic waters in 1966^5^, likely introduced through maritime transport^6^. Since its introduction, *A. kagoshimensis* has successfully established populations in various parts of the Mediterranean including the Catalan, Ligurian, Tyrrhenian, Adriatic, Aegean, Marmara, Black, and Azov seas^7^. *Anadara transversa* originates from the North-western Atlantic Ocean, with a native distribution ranging from Southern Massachusetts to Florida and Texas^8^. The species was initially reported in the Mediterranean Sea in Turkish waters in 1972^9^, with its first occurrence in the Adriatic Sea recorded in the 1970s^10^. Albano *et al*.^10^ suggested that *A. transversa* was most likely introduced through maritime activities, specifically via ballast water or attached to ship hulls. *Anadara transversa* has subsequently proliferated across multiple sectors of the Mediterranean^10,11^, including the Northern Aegean Sea^12^ and the Adriatic Sea^11,13–16^.

Within the Mediterranean, *A. kagoshimensis* and *A. transversa* are considered invasive species with reported moderate to high impacts on biodiversity and ecosystem services with major socio-economic consequences^3,17^, underscoring the critical need for monitoring and managing their presence in Mediterranean marine ecosystems. Indeed, invasive species such as *A. kagoshimensis*, which spawn during the summer, and *A. transversa*, which spawn year-round, present significant challenges for both native and cultivated bivalve species due to their competition for space^18^. *Anadara kagoshimensis* and *A. transversa* have significantly impacted marine ecosystem services in the Adriatic Sea. These impacts include adverse effects on food provision through fisheries and aquaculture^18,19^. Moreover, they have profoundly impacted the sandy infralittoral bottoms, which are a habitat for commercial species such as the venerid *Chamelea gallina* (Linnaeus, 1758)^20^.

The spatial distribution of *A. kagoshimensis* has been documented and monitored for four years in the Adriatic Sea (from 2008 to 2011 in Despalatović *et al*.^14^; and from 2010 to 2014 in Strafella *et al*.^2^), and for nearly one year within the biofouling communities associated with mariculture in the Northern Adriatic (Nerlović *et al*.^19^). However, systematic and extensive monitoring programmes, both in terms of geographic and temporal coverage, have often been lacking, or their associated data have remained inaccessible to the broader community. This is due to two main reasons. On one hand, so-called long-term monitoring efforts are frequently undermined by limited funding, insufficient institutional support, lack of recognition, poor communication, and competing priorities^21^. On the other hand, even when such programmes are implemented, they often fail to incorporate proper data management strategies or adopt open access policies to ensure data quality and broader usability^22^.

This dataset^23^ capitalises on by-catch records of *Anadara* spp. collected within the monitoring campaigns of the SoleMon project, an ongoing international initiative aimed at evaluating the population dynamics of commercially significant demersal species, notably *Solea solea* (Linnaeus, 1758), in the Central and Northern Adriatic Sea^24,25^. Data were collected through a modified beam trawl known as “rapido”, traditionally utilised by local fishers to harvest flatfish and other valuable benthic organisms. Beyond its principal focus, the SoleMon project gathers other by-catch information on several marine benthic species, both marketable and non-marketable, in addition to marine litter metrics (i.e., composition, density, and distribution). These data are instrumental in quantifying and evaluating the extent and ecological impact of marine debris, particularly plastics^26,27^, in marine ecosystems.

The SoleMon beam trawl surveys, conducted annually, provide a substantial source of information on the invasive *Anadara* spp., warranting proper extraction and utilisation to maximise their potential^28–30^. Spatiotemporal data on the abundance and distribution of these invasive bivalves can be crucial for designing effective management strategies. Additionally, when monitoring edible invasive species, as in the case of *Anadara* spp., their status can be assessed in relation to the Maximum Sustainable Yield (MSY) reference points to provide essential information to support harvesting programmes, one of the most effective management strategies for addressing aquatic invaders^31^.

## Methods

*Anadara* spp. records were collected in the Northern Adriatic Sea, designated as Geographical Sub-Area 17 (GSA 17) by the General Fisheries Commission for the Mediterranean (GFCM)^32^. This area falls within the Italian maritime jurisdiction and international waters (i.e., waters that lie outside the legal jurisdiction of any nation)^33^, covering over 378450 km². Data collection occurred during a total of 16 surveys within the framework of the SoleMon project. Beam trawl surveys were performed annually, from October to December, according to the methodology described in the SoleMon Handbook^34^. In brief, the trawling equipment consisted in a metallic rectangular frame, 3.59 m wide and 0.25 m tall, equipped with four skids and 46 iron teeth along its lower edge. An inclined wooden plank attached to the upper front of the frame ensured continuous contact with the seabed during operations. The gear included a polyamide net bag, with its lower section protected by a sturdy rubber diamond-mesh net attached to the frame. The net’s codend was 2.70 m long with a mesh size of 40 mm when stretched. At each station, the research vessel towed two trawling devices simultaneously to fish target species. A standard trawl typically lasted 30 minutes on average. However, in areas where certain species are highly abundant, some trawls were shortened to 10 minutes to prevent net saturation. These shorter trawls are followed by additional hauls, and the catches are pooled together.

The total catch from each trawl was measured using an electronic dynamometer (Dynafor LLX2) with a precision of ±3.2 kg. Biological subsamples of megazoobenthos were randomly collected from only one device’s haul. The percentage of megazoobenthic species was subsampled based on their weight: 100% for weights up to 30 kg, 50% for weights between 30 and 60 kg, 20% for weights between 60 and 500 kg, 10% for weights between 500 and 1500 kg, and 5% for weights exceeding 1500 kg. Megazoobenthic species were classified on board by taxonomic experts. Quantitative assessments, such as counting individual specimens (including *Anadara* spp.) and weighing them, were performed. The Raising factor was applied to scale the subsample to the entire haul (i.e., to the catch from both devices). SoleMon data including sampling effort (haul duration), sampling dates, depth, swept area, haul geographical coordinates (centroids), biomass (kg), and number of individuals, were archived in the database for Scientific Trawl Surveys [TruSt]^35^. The TruSt database compiles data from trawl surveys conducted by various research institutions and includes services for data storage, management, and analysis, such as the calculation of density and biomass per sampled area. The TruSt database, however, does not provide data and services open-access preventing the information stored therein to be used and exploited by a wider scientific community.

The opportunity to leverage distribution and abundance data of invasive *Anadara* spp., collected within the SoleMon surveys and archived in TruSt, was provided by the project USEit — Using operational synergies for the study and integrated management of invasive alien species in Italy. The project USEit, funded by the National Research Council of Italy (CNR), focuses on finding common strategies for alien species data collection and data management. Such strategies were tested through a number of use cases using existing data, including this dataset^23^, and novel data generated within the project. A sub-portal dedicated to USEit data and metadata was created and released within the LifeWatch Italy Data Portal. The USEit sub-portal offers accessibility to this dataset^23^ and other alien species data produced in USEit and published open-access according to international standards for biodiversity and ecological data and metadata^36,37^.

## Data Records

The data presented in this paper can be accessed at 10.48372/ZS4D-EM32. The DOI provides access to the metadata record published in the LifeWatch ERIC Metadata Catalogue and licensed under the Creative Commons public domain licence CC0. The dataset can be accessed and downloaded from the LifeWatch Italy Data Portal at https://data.lifewatchitaly.eu/handle/123456789/129593. The compiled dataset^23^ describes the spatiotemporal distribution and abundance of *Anadara* spp. in the Northern and Central Adriatic (GSA 17) for 16 years, and includes records of both detections and non-detections at sampled locations, acknowledging that non-detection does not necessarily imply true absence. Such data are crucial to unveil changes in the spatial structure of two invasive species and on their temporal dynamics.

**Fig. 1:**
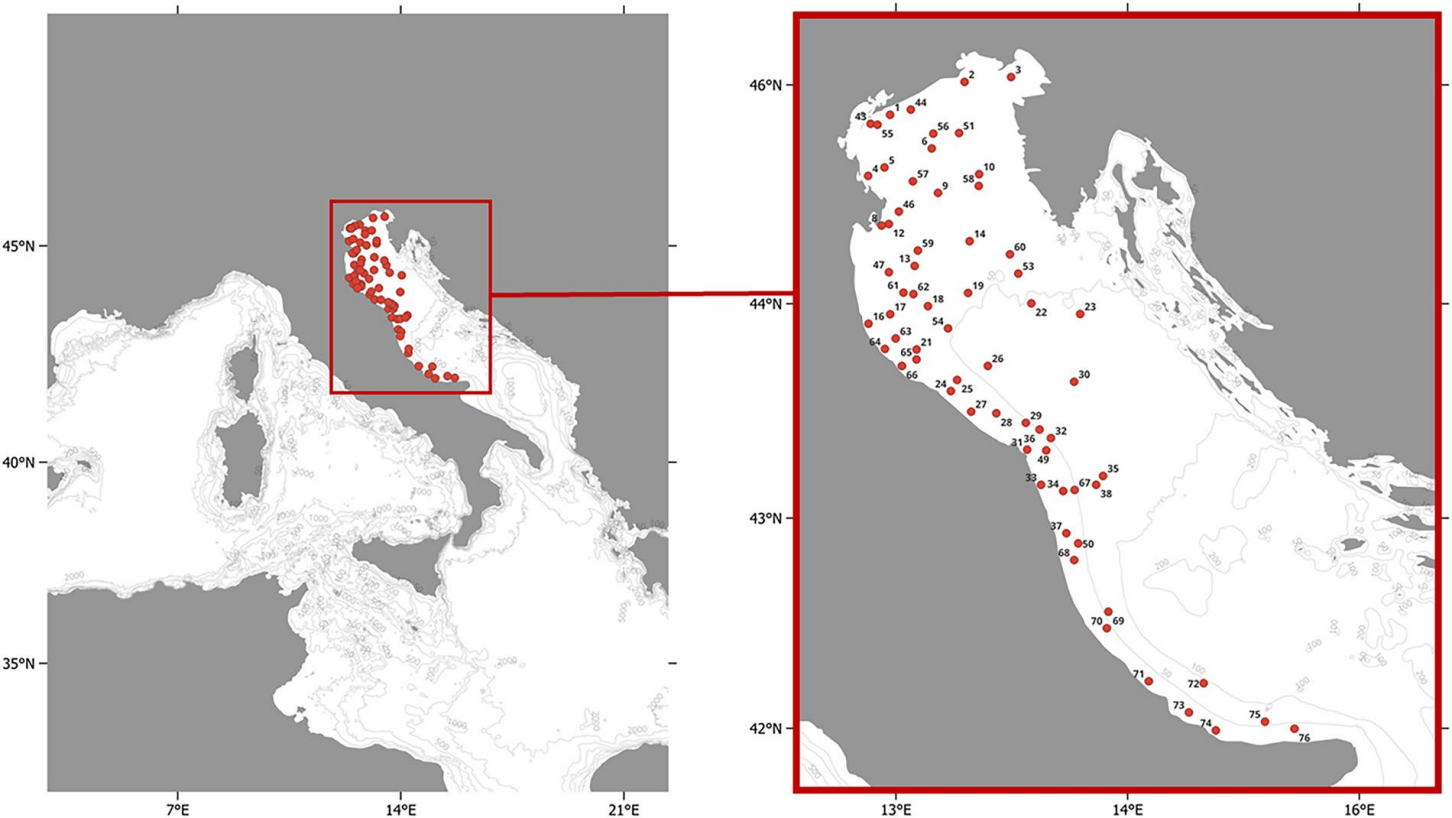
Map of the 66 SoleMon stations. Only stations located within Italian and international waters are included in this dataset and shown in the figure.

**Fig. 2:**
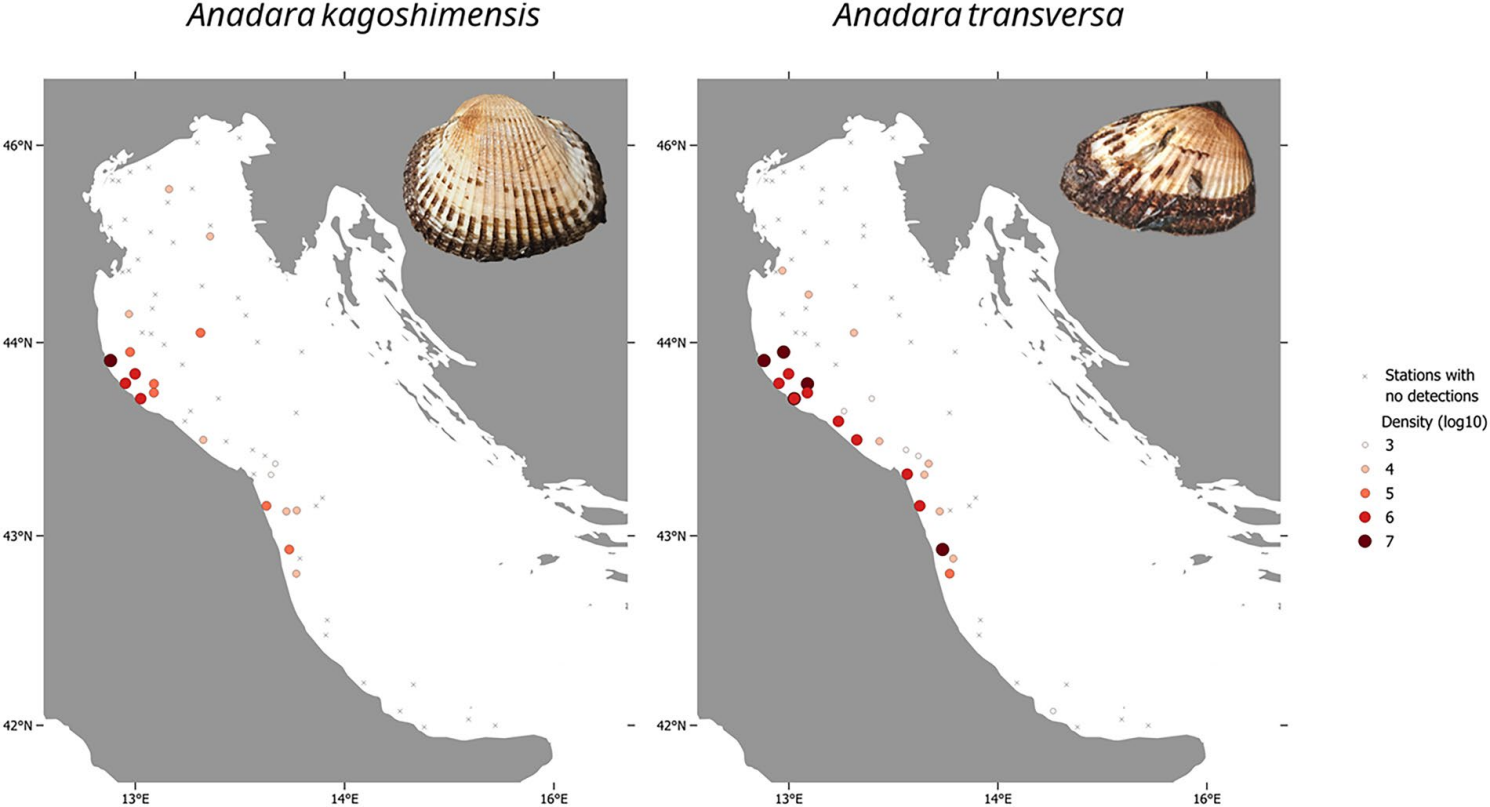
The distribution and density (i.e., number of individuals/km^2^) observed in 2023 of *Anadara kagoshimensis* (on the left) and of *A. transversa* (on the right) along with stations where no individuals were detected, across the 66 trawling stations included in the dataset.

**Fig. 3:**
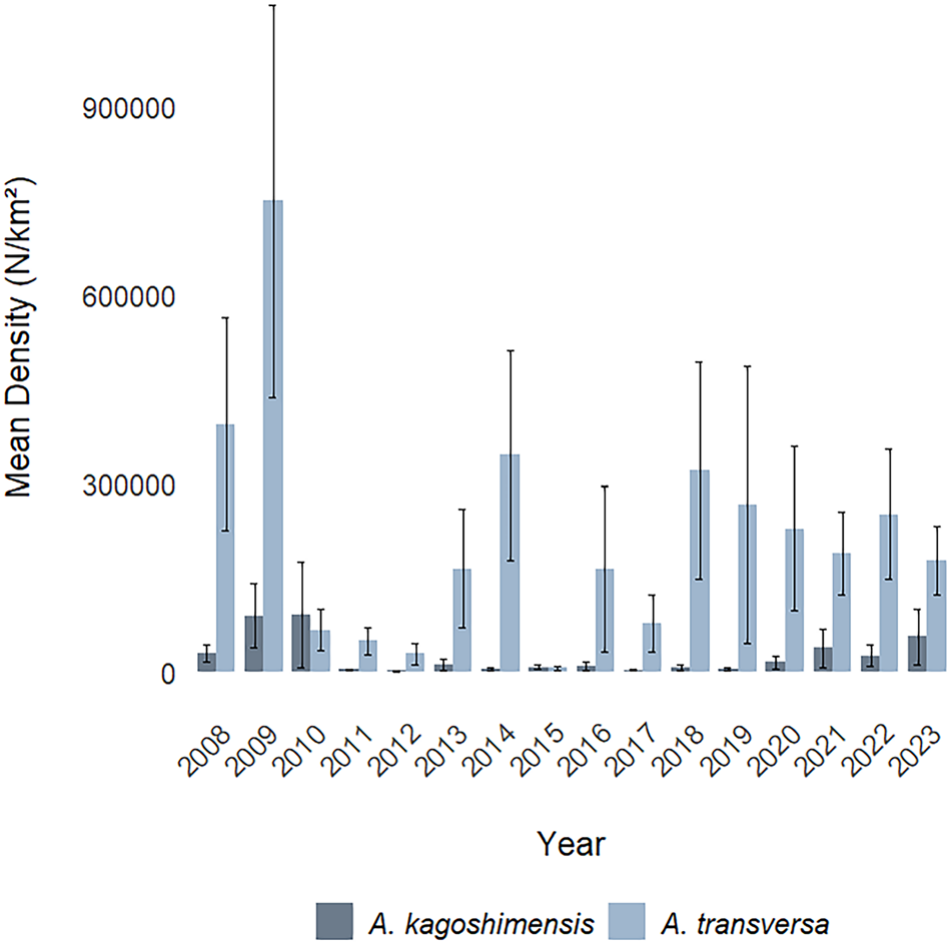
Mean density (i.e., average number of individuals per km² per year) with standard errors. Mean density was calculated using data from all 66 stations. Stations where the species were not detected are also illustrated.

The dataset includes 19 attributes (Table 1) describing geographic and temporal information (e.g., country, location, coordinates, depth, date), and sampling details (protocol, haul duration, swept area) of each record. In addition, population size indices associated with each record are provided including number of individuals (N), density (N/km²), wet weight biomass (kg), and biomass per unit area (kg/km²). In the 66 trawling stations considered in this dataset (Fig. 1), the biomass trends of *A. kagoshimensis* and *A. transversa*, along with size assessments (expressed as kg/N), distribution, and abundances, could be analysed annually over the 16-year period from 2008 to 2023. Figure 2 illustrates the distribution and density of *A. kagoshimensis* and *A. transversa* observed in 2023. Figure 3 shows the mean density per year across all stations, along with standard errors.

**Table 1:** The table describes the information included in the dataset.

Attribute label	Attribute IRI	Attribute description
catalogNumber	http://rs.tdwg.org/dwc/terms/catalogNumber	A unique identifier for the record within the dataset.
samplingProtocol	http://rs.tdwg.org/dwc/terms/samplingProtocol	A reference to the protocols used during the sampling event.
country	http://rs.tdwg.org/dwc/terms/country	The name of the country in which the sampling location occurs.
locationID	http://rs.tdwg.org/dwc/terms/locationID	An identifier of the sampling location (station).
depth	http://purl.dataone.org/odo/ECSO_00000515	The description of the depth below the sea surface.
samplingEffort	http://rs.tdwg.org/dwc/terms/samplingEffort	The amount of effort expended during the sampling event expressed as minutes of haul duration.
eventDate	http://rs.tdwg.org/dwc/terms/verbatimEventDate	The representation of the date information for the sampling event expressed as YYYY-MM-DD.
decimalLatitude	http://rs.tdwg.org/dwc/terms/decimalLatitude	The geographic latitude (in decimal degrees, using the spatial reference system WGS84) of sampling location (station).
decimalLongitude	http://rs.tdwg.org/dwc/terms/decimalLongitude	The geographic longitude (in decimal degrees, using the spatial reference system WGS84) of the sampling location (station).
taxonID	http://rs.tdwg.org/dwc/terms/taxonID	An identifier for taxon information within the dataset.
scientificName	http://rs.tdwg.org/dwc/terms/scientificName	The scientific name as originally provided by the dataset creator(s).
acceptedNameUsage	http://rs.tdwg.org/dwc/terms/acceptedNameUsage	The full scientific name, with authorship and date information, of the currently valid or accepted taxon.
acceptedNameUsageID	http://rs.tdwg.org/dwc/terms/acceptedNameUsageID	A unique identifier for the name usage according to the World Register of Marine Species (AphiaID).
sampleSizeValue	http://rs.tdwg.org/dwc/terms/sampleSizeValue	The numeric value for the measurement of the sampled area during the sampling event.
sampleSizeUnit	http://rs.tdwg.org/dwc/terms/sampleSizeUnit	The unit of measurement of the sampled area during the sampling event.
individualCount	http://rs.tdwg.org/dwc/terms/individualCount	The number of sampled individuals.
density	https://kos.lifewatch.eu/thesauri/traits/c_ddc0028f	The number of sampled individuals per unit area.
biomass	https://kos.lifewatch.eu/thesauri/traits/c_331cd569	The fresh weight of a biological sample including its water content and its organic and inorganic components.
biomassPerUnitArea	http://vocab.nerc.ac.uk/collection/P02/current/ZWTX/	The fresh weight biomass per unit area.

The column “Attribute label” lists the names of each attribute (dataset headers), the “Attribute IRI” column includes the Internationalized Resource Identifiers of the standard attributes, and the description column provides brief descriptions of each attribute.

Data collected by other projects were not included in this dataset, as the Italian partner of the project may, in the future, release updated versions of the dataset incorporating new data on *Anadara* spp. from the SoleMon project in both Italian and international waters.

## Technical Validation

Data curation was ensured through taxonomic and geographic validation. The taxonomic check was performed against the World Register of Marine Species^38^. QGIS version 3.34.4-Prizren was utilised to verify the accuracy of geographical coordinates, expressed in decimal degrees within the WGS84 reference system.

The data schema conforms to the Darwin Core standard^36^, and other controlled vocabularies from the NERC Vocabulary Server^39^ were used (Table 1). Metadata, available in the LifeWatch ERIC Metadata Catalogue, are described using the Ecological Metadata Language (EML) 2.2.0^37^. Metadata provides basic information (e.g., title, abstract, identifier), contacts, licence, and other technical information along with geographical, temporal, taxonomic descriptions, and data table information such as those described in Table 1.

## Usage Notes

Connection between data and metadata is ensured via the “Online Distribution” section of the metadata record and the “Alternate Identifier” section of the Data Portal.

The dataset could be combined with other biological data to assess the impact of *Anadara* spp. invasion on native communities within the Adriatic Sea at different temporal and spatial scales. The reuse of this dataset is facilitated by the metadata descriptions (e.g., sampling methods, adopted protocols), the information included in the dataset for each sampling event, *Anadara* spp. occurrence, and the standard labels (dataset headers) from controlled vocabularies. With the dataset now available and harmonised, and considering the ongoing nature of the project, future releases are likely to feature an increased number of records. This indicates the potential for the dataset to expand over time.

Moreover, this dataset could be integrated into other studies that examine the spatiotemporal trends of alien species in the same and/or different study areas. Such integration would enhance our understanding of the distribution patterns and ecological impacts of multiple alien and invasive species in the Mediterranean. This, in turn, would facilitate the development of regional management strategies to address the broader issue of biological invasions in marine ecosystems.

## Data Availability

Data visualisation was performed by using QGIS version 3.34.4-Prizren.
